# The role of antioxidants in facial nerve injury

**DOI:** 10.3389/fmolb.2025.1663998

**Published:** 2025-10-01

**Authors:** Jae Min Lee, Seong Wook Byun, Sung Soo Kim, Kyung Sun Park, Jae-Hong Ryoo, Seung Geun Yeo

**Affiliations:** ^1^ Department of Otorhinolaryngology Head and Neck Surgery, College of Medicine, Kyung Hee University Medical Center, Seoul, Republic of Korea; ^2^ Medical Research Center for Bioreaction to Reactive Oxygen Species and Biomedical Science Institute, Core Research Institute, Kyung Hee University, Seoul, Republic of Korea; ^3^ Department of Laboratory Medicine, College of Medicine, Kyung Hee University, Seoul, Republic of Korea; ^4^ Department of Occupational and Environmental Medicine, School of Medicine, Kyung Hee University, Seoul, Republic of Korea; ^5^ Department of Precision Medicine, Graduate School, Kyung Hee University, Seoul, Republic of Korea; ^6^ Department of Integrative Medicine, College of Medicine, Kyung Hee University, Seoul, Republic of Korea

**Keywords:** facial nerve injury, oxidative stress, antioxidants, reactive oxygen species, nerveregeneration

## Abstract

Oxidative stress contributes to the pathogenesis of facial nerve injury (FNI), yet the role of antioxidants in driving regeneration and functional recovery remains incompletely defined. This narrative review synthesizes studies published between 2008 and 2025 that evaluated antioxidant interventions in FNI across animal and limited human contexts. We systematically searched five databases and included 19 studies assessing oxidative stress markers and neural outcomes following antioxidant administration. To avoid overgeneralization, we stratified findings by injury model—crush/compression, transection with anastomosis, and ischemic/viral—and by primary endpoints (electrophysiology/behavior vs. histology/biochemistry), and, where reported, by intervention timing and dose. Antioxidants commonly reduced reactive oxygen species and modulated survival and inflammatory pathways, supporting neuroprotection and, in some models, improved electrophysiological or behavioral readouts. However, benefits varied by model and regimen: crush injuries showed earlier functional gains, whereas transection models more often demonstrated histological improvement without consistent short-term functional recovery; ischemic/viral studies frequently lacked standardized electrophysiological confirmation. Outcomes were also contingent on timing and dose (with earlier initiation and moderate dosing generally more favorable), and select combinations showed additive effects in preclinical settings. Overall, the evidence is predominantly preclinical, heterogeneous in dosing/timing/formulations, and limited by small sample sizes and inconsistent functional outcomes. Antioxidant strategies should therefore be considered hypothesis-generating rather than clinically recommendable at this time. Future research should use model-appropriate, standardized functional endpoints, prespecify timing/dose exploration, evaluate rational combinations, and conduct well-powered clinical trials to establish efficacy, optimal use, and safety.

## 1 Introduction

### 1.1 Facial nerve

The facial nerve, also known as the seventh cranial nerve, is a critical component of the cranial nervous system responsible for controlling the muscles involved in facial expression. The motor nerve fibers of the facial nerve are myelinated and include sensory, gustatory, and parasympathetic components. Anatomically, the facial nerve originates at the pontomedullary junction of the brainstem, passes through the internal auditory canal, and converges at the geniculate ganglion. This sensory ganglion is located within the facial canal and contains sensory nerve cell bodies that transmit taste from the anterior two-thirds of the tongue and sensation from the palate, auricle, and external auditory canal. After passing through the temporal bone and exiting the skull via the stylomastoid foramen, the facial nerve branches into small motor fibers extending to the posterior auricular nerve, auricular muscles, and stylohyoid muscle. It then enters the parotid gland and divides into the upper temporal and lower cervicofacial branches, further branching into five terminal branches: the temporal, zygomatic, buccal, marginal mandibular, and cervical branches. These branches control various facial-expression muscles, enabling movements such as raising the eyebrows, closing the eyes, smiling, and frowning. The facial nerve contains parasympathetic fibers that regulate salivary and lacrimal gland secretion, as well as gustatory sensory fibers for the anterior two-thirds of the tongue. This multifunctionality is essential for maintaining facial symmetry and various autonomic functions. Due to its complex anatomical pathways and diverse functions, the facial nerve is susceptible to injury from trauma, inflammation, and surgery, potentially leading to aesthetic and physiological issues such as facial asymmetry, dry eyes, and loss of taste. Understanding the anatomical and physiological complexity of the facial nerve is crucial for diagnosing and treating disorders related to facial nerve damage (Ho et al., 2015; Huang et al., 2023; Pauna et al., 2024; Seneviratne and Patel, 2025).

### 1.2 Facial nerve injury (FNI)

Facial nerve injury (FNI) significantly impacts a patient’s quality of life, leading to facial asymmetry that can complicate social interactions and cause psychological stress and social isolation. Additional physiological issues such as dry eyes, dry mouth, and speech difficulties can further diminish overall health and quality of life. FNI can result from various pathological conditions, including congenital malformation, trauma, inflammatory disease, tumors, and surgical interventions. Bell’s palsy, the most common cause of unilateral facial paralysis, occurs acutely and is often associated with viral infections. In most cases, partial or complete recovery from Bell’s palsy occurs within several months without treatment. FNI is prevalent in adults and closely associated with surgical procedures such as parotidectomy, mastoidectomy, and facial cosmetic surgeries. In adults, the prevalence of facial nerve paralysis is approximately 30 per 100,000 individuals annually. Post-injury, symptoms such as facial muscle weakness or paralysis can lead to facial asymmetry, difficulty closing the eyes, loss of nasolabial folds, and drooping of the mouth corner. The distal axons and myelin of the injured nerve undergo Wallerian degeneration (WD), characterized by the degeneration and disintegration of axons and myelin. Schwann cell proliferation and macrophage-mediated debris clearance create an environment conducive to nerve regeneration. The peripheral nervous system (PNS) has a faster myelin clearance rate and thus a higher regenerative capacity than the central nervous system (CNS). The regeneration capability of an axon may vary depending on the site and severity of the injury and the patient’s age. Cellular changes involved in axonal regeneration include Schwann cell activation, blood-nerve barrier disruption, and the recruitment of immune cells such as macrophages and cytokine-producing cells. Relevant molecular changes include metalloproteinase-mediated extracellular matrix modulation, neurotrophic factor upregulation, and cytokine production. These cellular and molecular changes create conditions that induce axon regeneration from the proximal nerve stump. Nerve injury also increases oxidative stress, exacerbating cellular damage and dysfunction. Oxidative stress results from the excessive production of free radicals (FR) and reactive oxygen species (ROS), which can damage cell membranes, proteins, and DNA. While the initial inflammatory response facilitates debris clearance and growth factor release, excessive or chronic inflammation can inhibit nerve regeneration. Therefore, therapeutic strategies after nerve injury should focus on precisely regulating the initial inflammatory response and oxidative stress to promote nerve regeneration. Given the complexity and prevalence of FNI and the limited availability of large-scale human studies, preclinical models, particularly small animal studies, play a crucial role in advancing our understanding of the mechanisms and potential therapeutic approaches. (Stoll and Müller, 1999; Cha et al., 2008; Gaudet et al., 2011; Nellis et al., 2017; Reich, 2017; Nocera and Jacob, 2020).

### 1.3 Oxidative stress

Oxidative stress occurs when there is an imbalance between the production of ROS and FR and the body’s ability to counteract their harmful effects through its antioxidant defense systems. ROS are chemically reactive molecules containing oxygen, including superoxide radicals (O_2_•−), hydrogen peroxide (H_2_O_2_), hydroxyl radicals (•OH), and singlet oxygen (^1^O_2_), naturally generated as byproducts of intracellular metabolic processes. Under normal physiological conditions, ROS play essential roles in cell signaling, immune response, and homeostasis. However, excessive ROS production can damage critical cellular structures, including cell membranes, proteins, and DNA, leading to cellular damage and dysfunction. This oxidative stress outcome can significantly exacerbate cellular damage and hinder nerve regeneration following FNI. The imbalance caused by excessive accumulation of ROS and FR leads to the severe degradation of essential biomolecules such as proteins, lipids, and DNA, ultimately impairing cellular function and recovery processes. Oxidative damage is particularly harmful to neurons, as it leads to mitochondrial dysfunction, decreased ATP production, and depletion of the energy necessary for cell survival. Mitochondrial dysfunction can further increase ROS production, creating a vicious cycle of oxidative stress. ROS can also induce lipid peroxidation of cell membranes, compromising their structural integrity and function. This damage can trigger inflammatory signaling pathways. While the initial inflammatory response is crucial for clearing debris and initiating healing, chronic inflammation can lead to further damage and delayed recovery. Excessive ROS generated during neuroinflammatory processes can induce apoptosis or necrosis of nerve cells, reducing the number of viable cells necessary for regeneration. ROS can also alter the signaling pathways of growth factors essential for nerve regeneration, further hindering the regenerative process. Although ROS are primarily generated in mitochondria, they can also arise from enzymatic activities in pathways such as cellular respiration and arachidonic acid metabolism. Cells possess intrinsic antioxidant systems involving enzymes such as superoxide dismutase (SOD), catalase (CAT), and glutathione peroxidase (GPx), which neutralize ROS. However, this system often fails to fully manage high ROS levels, leading to oxidative stress. The use of antioxidant therapies aimed at mitigating oxidative stress may be a promising strategy to promote peripheral nerve recovery. Preclinical studies provide essential insights into the oxidative stress mechanisms and the potential efficacy of antioxidant interventions, which are crucial for developing future clinical applications (Pizzino et al., 2017; Juan et al., 2021; Adamu et al., 2024).

### 1.4 Facial nerve injury and oxidative stress

FNI can arise from various causes, including viral and bacterial infection, cerebral infarction, trauma, tumors, and surgery. When the facial nerve is damaged, an active anterograde degeneration process called Wallerian degeneration (WD) occurs at the distal ends of the terminal branches, leading to increased ROS production and oxidative stress. After FNI, ROS and free radicals (FR) significantly affect various intracellular signaling pathways that complexly regulate physiological processes such as apoptosis, inflammatory responses, and cell survival. Facial nerve transection inhibits tubulin polymerization and disrupts microtubule assembly, hindering nerve regeneration. Reactive species such as nitric oxide (NO) and peroxynitrite oxidize polyunsaturated fatty acids, accumulate in neuronal membranes, and produce toxic aldehydes that compromise membrane integrity and function. Inhibition of NO and peroxynitrite can promote axonal regeneration and recovery after peripheral nerve injury (PNI). Increased ROS levels activate glial cells, such as microglia and astrocytes, which play a dual role in the injury response. These cells secrete pro-inflammatory cytokines [e.g., interleukin-1 beta (IL-1β) and tumor necrosis factor-alpha (TNF-α)] that can exacerbate inflammation and accelerate neurodegeneration. Overactivated glial cells release neurotoxic mediators that worsen neuroinflammation and cause further neuronal damage. However, glial cells also contribute to the antioxidant defense by producing enzymes that neutralize ROS and by secreting neurotrophic factors that support axonal regeneration and remyelination. Therefore, reducing excessive glial cell activation and inflammation while enhancing their neuroprotective functions is crucial for promoting nerve recovery. Human herpesvirus 7 (HHV7) infection increases ROS production, leading to facial nerve damage and oxidative stress through increased malondialdehyde (MDA) levels and decreased superoxide dismutase (SOD) activity. These findings underscore the need for integrated therapeutic strategies that regulate FR and ROS, enhance mitochondrial health, and control inflammation to improve nerve injury recovery. Efforts to comprehensively address these oxidative processes will be essential for developing effective treatments aimed at addressing FNI and improving patient outcomes. This review emphasizes preclinical models, specifically small animal studies, due to their prevalence in the literature and their ability to provide detailed insights into the oxidative processes and therapeutic potential of antioxidants in FNI. These models are instrumental in elucidating the underlying mechanisms and guiding the development of effective treatment strategies for FNI (Chang et al., 2021; Yeh and Liu, 2021; Oh et al., 2024; Lee et al., 2025).

## 2 Research methods

**FIGURE 1:**
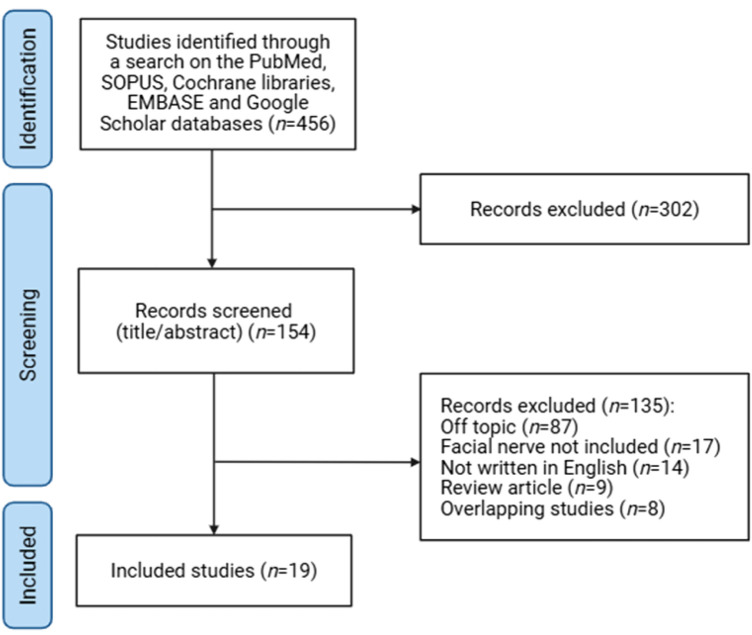
Review flow diagram.

Although extensive research has examined the role of antioxidants in facial nerve injury (FNI) and various diseases, the field has lacked comprehensive reviews specifically addressing the impact of antioxidant therapy on nerve regeneration post-injury. To fill this gap, one of the authors (S.W.B.) conducted a thorough literature search across five electronic databases—Cochrane Libraries, EMBASE, Google Scholar, PubMed, and SCOPUS—covering publications from January 2008 to May 2025. Keywords such as “facial nerve injury” and “antioxidants” were used in the search, which focused on studies published in English and included both human and animal studies investigating the association between FNI and antioxidants. This search strategy retrieved a total of 154 studies. Exclusions included studies not related to the topic (87), studies not involving the facial nerve (17), non-English publications (14), review articles (9), and duplicate studies (8). Ultimately, 19 studies were selected (Figure 1) and analyzed for information on changes in oxidative stress and nerve regeneration following post-FNI antioxidant administration. This review aims to provide insights into the potential therapeutic efficacy of antioxidants on FNI by synthesizing their impact on the pathogenesis of facial nerve degeneration and regeneration (Figure 1). The literature search was designed to focus on preclinical studies, with an emphasis on small animal models, to investigate the effects of antioxidant treatments on facial nerve injury. This focus was chosen due to the limited availability of large animal and human studies in this area and the critical insights these models provide into the efficacy and mechanisms of antioxidant therapies.

## 3 Results

### 3.1 Oxidative stress factors associated with facial nerve injury

Facial nerve injury (FNI) is associated with an increase in oxidative stress, which plays a critical role in nerve degeneration. Experimental models of FNI, induced through various methods such as nerve transection, compression, and viral infection, show a significant increase in oxidative stress markers and a decrease in antioxidant substances (Table 1). This oxidative stress is characterized by elevated levels of superoxide radicals (O_2_·−), hydrogen peroxide (H_2_O_2_), hydroxyl radicals (·OH), nitric oxide (NO), nitric oxide synthase (NOS), NADPH oxidase 2, peroxynitrite (ONOO−), malondialdehyde (MDA), and 4-hydroxynonenal (4-HNE). Concurrently, there are decreases in key antioxidants such as glutathione (GSH), superoxide dismutase (SOD), catalase (CAT), glutathione peroxidase (GPx), and glutathione peroxidase 4 (GPX4).

**TABLE 1:** Changes in oxidative stress factors and antioxidant substances following FNI.

Factors	Substances
Increased oxidative stress factors	Superoxide Radical (O_2_⋅−) Hydrogen Peroxide (H_2_O_2_) Hydroxyl Radical (⋅OH) Nitric Oxide (NO) Nitric Oxide Synthase (NOS) NADPH oxidase 2 Peroxynitrite (ONOO−) Malondialdehyde (MDA) 4-Hydroxynonenal (4-HNE)
Decreased antioxidant substances	Glutathione (GSH) Superoxide Dismutase (SOD) Catalase (CAT) Glutathione Peroxidase (GPx) Glutathione Peroxidase 4 (GPX4)

### 3.2 Antioxidants effective against facial nerve injury

The present review of 19 studies indicates that the administration of antioxidants is associated with functional recovery, improved nerve conduction, and increased levels of histological markers for nerve recovery. Facial nerve regeneration is a complex process influenced by oxidative stress, inflammation, apoptosis, and cellular metabolic disturbances following nerve injury. Antioxidants intervene at various points in these pathological cascades to create an environment conducive to regeneration. Their mechanisms of action include direct scavenging of ROS, modulation of survival and recovery-related signaling pathways, stabilization of mitochondria, and support of neurotrophic activity. These actions contribute to maintaining nerve integrity, promoting axonal regrowth, and enhancing Schwann cell remyelination (Table 2).

**TABLE 2:** FNI and the efficacy of antioxidants.

Author/Year	Injury method	Species and/or sample	Target substances/dosage	Delivery/recovery timeline	Detection method	Mechanism of action	Results/Conclusions
Yildirim et al. (2015)	Crush	Sprague-Dawley rats (n = 16) - Control group (n = 8): Crush injury + saline - Crush injury + coenzyme Q10 (n = 8)	CoQ10 (10 mg/kg/day)	CoQ10 administered post-injury via intraperitoneally injection for 30 days /1 month	Electrophysiology (EMG), Histology	Reduces oxidative stress and promotes nerve repair	One month after treatment, the CoQ10 group had significantly lower stimulation thresholds, indicating better nerve function compared to the control group. Nerves treated with CoQ10 showed significantly less vascular congestion and macrovacuolation than those in the control group. The CoQ10-treated group exhibited greater myelin thickness compared to the control group /Administration of CoQ10 improved the functional and histological recovery of crushed facial nerves. The CoQ10 group demonstrated faster electrophysiological recovery, suggesting enhanced nerve conduction. The histological improvements in the CoQ10 group suggest that this treatment reduces oxidative stress-induced secondary nerve damage
Tekdemir et al. (2018)	Crush	Wistar rats (n = 30) - Control group (n = 6): No trauma - Crush injury + lipoic acid (LA) (n = 8) - Crush injury + methylprednisolone (MP) (n = 8) - Crush injury + LA + MP (n = 8)	LA (100 mg/kg) MP (1 mg/kg/day)	Intraperitoneal injection for 1 week post-injury /1 week	EMG	Antioxidant effects; reduces oxidative stress and enhances nerve regeneration	Combined treatment with lipoic acid (LA) and methylprednisolone (MP) yielded significantly lower nerve stimulus thresholds compared to LA treatment alone, indicating that nerve function was better in the co-treated group. While the LA and MP mono-treatment groups had higher post-treatment thresholds than the control group, the LA + MP combination group showed no significant difference from the control, suggesting that nerve function recovery was comparable in the latter two groups. Histological examination revealed improved nerve regeneration and reduced inflammation across all treatment groups, with the LA + MP group demonstrating the most significant improvements /LA positively influences nerve healing and enhances the effects of MP. The combination of LA and MP is more effective than either treatment alone, indicating a synergistic effect. LA may serve as a viable alternative in scenarios where MP is not suitable for use
Yoo et al. (2022)	Crush, Axotomy	Sprague-Dawley rats (n = 40) - Crush injury only (n = 10) - Crush injury + alpha lipoic acid (ALA) (n = 10) - Axotomy only (n = 10) - Axotomy + ALA (n = 10)	ALA (20 mg/kg/day)	Intraperitoneal injection immediately after injury /2 weeks	Immunohistochemical, Behavioral tests	Acts as an antioxidant, reducing oxidative stress and promoting nerve regeneration	Nicotinamide adenine dinucleotide phosphate oxidase 2 (NOX2) expression was higher in axotomy injury compared to crush injury. ALA treatment significantly increased NOX2 expression in the axotomy group. Behavioral tests showed better recovery in the crush injury + ALA group; this was particularly notable in the improved eye blink reflex scores seen on day 4 post-injury /ALA can promote nerve regeneration in crush injury models and significantly increase NOX2 expression after nerve injury. ALA administration may be a promising strategy for accelerating recovery from peripheral FNI, particularly in cases of crush injury
Şeneldir et al. (2024)	Crush	Wistar rats (n = 30) - Control group (n = 8): crush injury, no treatment - Sham group (n = 6): no crush injury - Crush injury + Ebselen (n = 8) - Crush injury + methylprednisolone (n = 8)	Ebselen (10 mg/kg/day) Methylprednisolone (1 mg/kg/day)	Oral administration for 10 days /3 weeks	EMG, Histology, Behavioral tests	Antioxidant and neuroprotective effects; reduces oxidative stress and enhances nerve regeneration	The ebselen and MP groups showed similar recoveries, with faster improvements in eye blink reflexes and vibrissae movement compared to the control group. Electrophysiological assessments confirmed comparable effectiveness in nerve regeneration. Histopathological analysis revealed improvements in axonal degeneration and myelin thickness, although macrovacuolation remained unchanged. Ebselen demonstrated a similar or better effect compared to MP. /Ebselen is as effective as MP in promoting recovery from facial nerve crush injury, offering a promising alternative with fewer side effects. Ebselen’s neuroprotective and antioxidant properties contribute to the observed functional and structural recoveries
Tanyeri et al. (2015)	Crush	Wistar rats (n = 40) - Control group (n = 8): No treatment after crush injury - Crush injury + physiological serum (n = 8) - Crush injury + dexamethasone (n = 8) - Crush injury + oxytocin (n = 8) - Crush injury + resveratrol (n = 8)	- Dexamethasone (2 mg/mL) - Oxytocin (40 mg/kg) - Resveratrol (30 mg/kg)	Intraperitoneal injection for 28 days /8 to 12 days	EMG, Histology, Immunohistochemistry, Behavioral tests	Antioxidant and neuroprotective effects	The recovery time of facial reflexes after injury was shortest in the resveratrol group. The peak-to-peak amplitude values ​​were significantly increased in the resveratrol, dexamethasone, and oxytocin groups, and this increase was more pronounced in the resveratrol group. Nerve regeneration was most evident in the resveratrol group. Moderate apoptotic changes were observed in the resveratrol and oxytocin groups, as assessed by TUNEL staining and antibodies against caspase 3 and -6. Connexin 32 and -43 were upregulated in the resveratrol group /Resveratrol yields the best results after facial nerve compression injury, and dexamethasone and oxytocin also significantly improve nerve regeneration. Resveratrol and oxytocin may be useful in treating FNI patients
Karlidag et al. (2012)	Axotomy	New Zealand white rabbits (n = 30) - Control group (n = 10): No treatment after facial nerve anastomosis - Anastomosis + N-acetylcysteine (NAC) (n = 10) - Anastomosis + methylprednisolone (MP) (n = 10)	NAC (50 mg/kg/day) Methylprednisolone (1 mg/kg/day)	Intramuscular injection for 2 months /2 months	EMG, Histology	Antioxidant effects; enhances nerve regeneration	The group treated with N-acetylcysteine (NAC) achieved the most effective nerve regeneration, largely due to increased proliferation of Schwann and glial cells, while the MP group showed the weakest regeneration. Degeneration was most pronounced in the MP group, with significant axonal withdrawal and increased myelin debris, whereas the NAC group exhibited the least degeneration. Microscopic analysis confirmed that tissue healing was better in the NAC group compared to the control, while the MP group showed notable degeneration characterized by increased fibrosis and collagen fibers. The MP group had significantly more myelin debris, highlighting its greater degeneration /MP does not aid in nerve regeneration after facial nerve anastomosis; rather, it increases degeneration. Conversely, NAC significantly improves nerve regeneration and reduces degeneration, and thus may serve as an effective adjunct to surgical treatment for enhancing nerve healing following traumatic facial nerve paralysis
Rivera et al. (2017)	Crush	Wistar rats (n = 24) - Control group (n = 12): No treatment after facial nerve crush - Crush + N-acetylcysteine (NAC) (n = 12)	N-acetyl-cysteine (50 mg/kg/day via subcutaneous osmotic pump)	Evaluated at 2 and 4 weeks post-injury /4 weeks	EMG, Functional testing	NAC acts as an antioxidant, reducing oxidative stress and cellular damage, promoting neuronal survival and regeneration	NAC treatment yielded significantly better recovery of facial nerve function after a crush injury in rats. Specifically, rats treated with NAC showed better recovery in terms of eye blinking and vibrissae movement compared to the control group at both 2 and 4 weeks post-injury. Electromyographic (EMG) measurements indicated that NAC-treated rats had greater amplitude recovery, particularly for the eye blink function at 2 weeks and vibrissae function at 4 weeks post-injury. These results suggested that NAC can enhance the functional and electrical recovery of nerves following injury /NAC facilitates more effective and faster recovery of facial nerve function after crush injury, likely due to its antioxidant and neuroprotective effects. NAC may thus hold promise as a therapeutic agent for nerve injuries; further research is needed to explore its mechanisms and compare its efficacy with those of other treatments
Salomone et al. (2018)	Axotomy	Wistar rats (n = 60) - Microsuture repair only (24 h delay) (n = 15) - Microsuture + polyethylene glycol (PEG)-fusion (24 h delay) (n = 15) - Microsuture repair only (72 h delay) (n = 15) - Microsuture + PEG-fusion (72 h delay) (n = 15)	Polyethylene glycol solution (500 mM) with methylene blue (100 μM)	Microsuture performed after 24 and 72 h, followed by PEG-fusion protocol /6 weeks	EMG, Histology	PEG promotes axonal membrane fusion, while methylene blue prevents premature sealing as an antioxidant	At 6 weeks post-surgery, groups receiving the polyethylene glycol (PEG) fusion protocol showed lower latency and duration of nerve conduction compared to the control groups, indicating that the treatment may have slowed demyelination. PEG fusion-treated neurons had larger axonal diameters compared to controls, suggesting that this treatment may help preserve axonal structure. There was a high mortality rate among rats subjected to the procedures, particularly in groups undergoing two surgeries within short time intervals /PEG fusion does not significantly enhance functional recovery, but appears to slow demyelination and increase axonal diameter. The timing of surgical intervention, whether at 24 or 72 h post-injury, does not significantly affect the functional outcomes in severe nerve lesions. PEG fusion might be beneficial when applied immediately after trauma, as axonal membrane regeneration heavily depends on immediate post-injury conditions. Combination treatment with PEG fusion plus antioxidants may offer a promising approach for treating FNI.
Edizer et al. (2019)	Axotomy	Wistar albino rats (n = 50) - Control group (n = 10): Axotomy and neurorrhaphy, no treatment - Axotomy + topical melatonin (n = 10) - Axotomy + systemic melatonin (n = 10): - Axotomy + topical dexamethasone (n = 10) - Axotomy + systemic dexamethasone (n = 10)	Melatonin (20 mg/mL topically, 20 mg/kg systemically) Dexamethasone (4 mg/mL topically, 1 mg/kg systemically)	Evaluated at 3, 6, 9, and 12 weeks post-administration /12 weeks	EMG, Histology	Melatonin may induce Schwann cell proliferation and reduce collagen deposition, aiding nerve regeneration; Dexamethasone has anti-inflammatory effects	By the end of the 12-week study, the groups treated with melatonin showed superior histopathological outcomes compared to other groups. These outcomes included larger nerve diameters and the preservation of normal myelin ultrastructure, indicating that melatonin had a protective effect against myelin degeneration. In contrast, the control group exhibited significant inflammatory cell infiltration and more extensive myelin degeneration. While the dexamethasone-treated groups also demonstrated some positive histopathological effects, these were less pronounced than those observed in the melatonin-treated groups. Despite these structural improvements, electrophysiological assessments conducted over the 12-week period did not show significant improvements in nerve function, such as amplitude and latency of nerve conduction, for any of the treatment groups compared to preoperative levels /The study concluded that melatonin might significantly enhance facial nerve regeneration following axotomy and end-to-end anastomosis, as evidenced by its beneficial histopathological effects. While both melatonin and dexamethasone have potential roles in supporting nerve regeneration, the electrophysiological evidence of functional recovery might take longer than the 12-week study period to become apparent. Therefore, although structural regeneration is evident, functional improvements in nerve conduction may require additional time to manifest
Yanilmaz et al. (2015)	Axotomy	New Zealand white rabbits (n = 32) - Control group (saline) (n = 8) - Axotomy + aminoguanidine (AG) (n = 8) - Axotomy + melatonin (n = 8) - Axotomy + methylprednisolone (MP) (n = 8)	Aminoguanidine (100 mg/kg/day intraperitoneally for 14 days) Melatonin (30 mg/kg/day intraperitoneally for 10 days) Methylprednisolone (1 mg/kg/day intramuscularly, tapering dose)	Evaluated at 3, 6, and 10 weeks post-surgery /10 weeks	Nerve conduction studies (NCS), Histological analysis	AG acts as an antioxidant, reducing free radicals and myelin degeneration; Melatonin is also an antioxidant and reduces inflammation; Methylprednisolone is a corticosteroid with anti-inflammatory effects	Aminoguanidine (AG) yielded significant nerve regeneration by reducing myelin degeneration and myelin debris accumulation. AG also increased nerve conduction velocity and amplitude over time, indicating that it improved neural function. Melatonin effectively reduced collagen fiber enhancement and myelin degeneration compared to the control group. While melatonin yielded limited recovery with decreased nerve conduction latency, it did not result in a significant change in signal amplitude. In contrast, MP did not significantly improve nerve regeneration compared to the control group. MP prevented the increase in collagen fibers, but did not enhance Schwann cell proliferation or overall nerve function /Both aminoguanidine and melatonin enhanced nerve regeneration following peripheral facial nerve neurorrhaphy, as evidenced by histopathological and electrophysiological improvements. Aminoguanidine, in particular, showed the most promising results. In contrast, methylprednisolone did not demonstrate significant benefits in nerve recovery. These findings suggest that aminoguanidine and melatonin could be potential therapeutic agents for treating traumatic peripheral facial nerve palsy, but further studies are needed to evaluate their efficacy in humans
Koyama et al. (2022)	Crush	C57Bl/6J mice (n = 40) - Control group (n = 8): Crush injury, no treatment - Crush injury + Si-based agent (n = 8) - Crush injury + methylcobalamin (MeCbl) (n = 14) - Crush injury + Si-based agent + MeCbl (n = 10)	Si-based agent: 2.5% in diet MeCbl: 3% in electrospun nanofiber sheet	Evaluated over 22 days post-injury /22 days	EMG, Immunofluorescent staining, Oxidative stress measurement	Si-based agent generates antioxidant hydrogen, reducing oxidative stress; MeCbl promotes nerve regeneration	An Si-based agent improved clinical scores for facial paralysis by promoting myelination and reducing oxidative stress. Methylcobalamin (MeCbl) enhanced nerve regeneration and further reduced oxidative stress, with particularly notable effects when combined with the Si-based agent. Combination therapy with the Si-based agent and MeCbl was more effective than either treatment alone, significantly accelerating recovery and reducing the time needed for functional improvement /Together, the Si-based agent and MeCbl offer a promising therapeutic strategy for treating facial paralysis by promoting nerve repair and reducing oxidative stress. This combination therapy could improve prognosis and treatment outcomes for patients with intractable facial paralysis, thereby enhancing their quality of life
Ozbay et al. (2017)	Crush	Wistar albino rats (n = 14) - Control group (n = 7): Crush injury, no treatment - Crush injury + ozone therapy (n = 7):	Ozone: 1.1 mg/kg/day, intraperitoneally for 30 days Control: 1.1 mg/kg/day saline, intraperitoneally for 30 days	Evaluated before crush, immediately after, and 30 days post-crush /30 days	EMG, Light and electron microscopy	Ozone induces moderate oxidative stress, enhancing endogenous antioxidant systems and promoting nerve regeneration	The ozone-treated group exhibited somewhat lower stimulation thresholds compared to the saline group, suggesting that the former yielded greater functional improvement; however, the difference was not statistically significant. Histopathological evaluations revealed significant differences between the ozone and control groups in terms of vascular congestion, macrovacuolation, and myelin thickness. Electron microscopy confirmed structural improvements in the ozone group, which showed more normal myelinated axons and less damage compared to the control group /The study suggests that ozone therapy may have beneficial effects on the regeneration of crushed facial nerves, as evidenced by improved histopathological outcomes. Thus, ozone therapy could be a promising treatment avenue for acute facial paralysis and peripheral nerve regeneration. However, further studies with longer follow-up periods are needed to confirm these results
Dincer et al. (2025)	Crush	Sprague-Dawley rats (n = 28) - Control group (n = 7): No treatment - Crush injury + methylprednisolone (MP) (n = 7) - Crush injury + rosuvastatin (RSV) (n = 7) - Crush injury + RSV + MP (n = 7)	Methylprednisolone: 1 mg/kg/day, intraperitoneally Rosuvastatin: 10 mg/kg/day, oral gavage	Evaluated over 21 days post-injury /21 days	Behavioral tests, EMG, Histology	Rosuvastatin has antioxidant, neuroprotective, and anti-inflammatory effects, enhancing nerve regeneration	On day 21 post-treatment, the electrophysiological threshold values in the rosuvastatin and combination groups were significantly lower than that in the control group, indicating that the experimental treatments yielded better nerve function. Corneal reflex recovery was faster in the rosuvastatin and combination groups compared to the control and MP groups. Rosuvastatin + MP treatment was associated with significantly less axonal degeneration and vascular congestion than seen in the other groups /Rosuvastatin, especially when combined with MP, significantly improves facial nerve regeneration in terms of electrophysiological, functional, and histopathological recovery. This combination therapy shows promise for treating traumatic peripheral facial paralysis by enhancing nerve regeneration and accelerating motor function recovery. Further studies are needed to determine the optimal dosing and compare the efficacy of this strategy to those of other treatments
Jang et al. (2012)	Crush	Sprague-Dawley rats (n = 20) - Control group (n = 10): No treatment - Crush injury + ginkgo biloba extract (GBE) (n = 10)	GBE: 4 mL/kg, intraperitoneally, 30 min before injury and every other day for 4 weeks	Evaluated over 4 weeks post-injury /4 weeks	Behavioral tests, EMG	GBE has antioxidant activity, protecting vascular endothelial cells from oxidative injury and promoting nerve regeneration	The administration of ginkgo biloba extract (GBE) significantly accelerated the recovery of vibrissae function in the experimental group compared to the control group. Rats treated with GBE exhibited an earlier return to normal vibrissae movements and orientation, indicating enhanced functional recovery following nerve injury. By week 4 post-surgery, the experimental group showed significant improvements in muscle action potential thresholds and conduction velocities. This suggests that GBE treatment facilitated better electrophysiological recovery of the injured facial nerve /GBE effectively promotes nerve regeneration and functional recovery in a rat model of facial nerve crush injury. The observed benefits likely reflect the antioxidant properties of GBE, which contribute to its neuroprotective effects
Hoshida et al. (2009)	Axotomy	Sprague-Dawley rats (n = 36) - Axotomy +0.1% DMSO (n = 12) - Axotomy +20 μg/mL of vitamin E (n = 12) - Axotomy +200 μg/mL of vitamin E (n = 12)	Vitamin E: 20 mg/mL (moderate) or 200 mg/mL (high), in drinking water	Evaluated at 1, 2, and 4 weeks post-injury /4 weeks	Histology, Immunohistochemistry	Vitamin E acts as an antioxidant, reducing oxidative stress and lipid peroxidation, thus protecting motor neurons	A moderate dose of vitamin E (20 μg/mL) significantly increased the survival of motor neurons in the facial motor nucleus (FMN) compared to controls. A high dose of vitamin E (200 μg/mL) also improved survival but to a lesser extent. The moderate dose of vitamin E reduced gliosis, whereas the high dose increased glial cell proliferation. Vitamin E treatment also suppressed the expression of 4-hydroxynonenal (HNE), indicating that the vitamin exerts its antioxidant effects by reducing lipid peroxidation and oxidative stress /Vitamin E demonstrates neuroprotective effects by decreasing oxidative stress and enhancing neuronal survival following FNI. The study emphasizes the importance of proper dosing, as the moderate dose was more effective than the high dose in reducing oxidative damage and promoting recovery
Gölcük and Şeneldir (2025)	Crush	Sprague Dawley rats (n = 20) - Control group: No treatment - Crush injury + caffeic acid phenethyl ester (CAPE) - Crush injury + methylprednisolone - Crush injury + CAPE + methylprednisolone	CAPE: 10 μmol/kg/day Methylprednisolone: 1 mg/kg/day	Evaluated over 3 weeks post-injury /3 weeks	Behavioral test, EMG, Histology	CAPE has antioxidant, anti-inflammatory, and neuroprotective properties, enhancing nerve regeneration	Groups treated with caffeic acid phenethyl ester (CAPE) and CAPE + MP showed significant improvements in nerve excitability thresholds and histopathological recovery compared to the control group. The CAPE and CAPE + MP groups exhibited faster functional recovery in terms of corneal reflex and a more significant improvement in histopathological evaluations after 3 weeks. Specifically, these groups demonstrated lower levels of axonal degeneration and vascular congestion and greater myelin sheath thickness than the control group /CAPE + MP effectively promoted nerve regeneration in an experimental rat facial nerve crush model. The study suggests that CAPE exerts neuroprotective, antioxidant, and anti-inflammatory properties, and may serve as a potential therapeutic alternative to reduce the dosage and mitigate the systemic adverse effects of MP.
Pothion et al. (2022)	Axotomy	Long–Evans rats (n = 27) - Epineural suture (n = 10) - Axotomy + control (n = 10): - Axotomy + SELENOT mimetic peptide (PSELT) (n = 10): Nerve segments bridged with a femoral vein graft, injected with PSELT	PSELT: 1 μM, injected at injury site immediately and 48 h post-injury	Evaluated over 12 weeks post-injury /3 months	Behavioral test, EMG, Histology	PSELT, a SELENOT-derived peptide, has antioxidant and neuroprotective properties, enhancing nerve myelination and regeneration	The SELENOT-derived peptide 43–52 (PSELT) treatment yielded significantly improved motor recovery compared to the control and standard epineural suture groups. Specifically, PSELT treatment resulted in greater protraction amplitude and velocity of vibrissae movement, indicating enhanced functional motor recovery. Electromyographic analysis revealed that the PSELT group had a higher average motor unit action potential (MUAP) amplitude and shorter MUAP duration, suggesting improved nerve regeneration. Histologically, PSELT-treated rats exhibited increased axonal density and myelination, with a higher proportion of myelinated axons and more Schwann cells compared to controls /PSELT treatment significantly enhances nerve regeneration and functional recovery after PNI. By promoting axonal growth and myelination, PSELT offers a novel therapeutic approach for treating PNI that surpasses the outcomes of traditional surgical interventions
Hato et al. (2013)	Herpes simplex virus type 1 (HSV-1) infection	Balb/cAjcl mice (n = 62) - HSV-1 (n = 32) - HSV-1 + edaravone (n = 30)	Edaravone: 1 mg/kg or 10 mg/kg/day, intraperitoneally for 11 days	Evaluated over 14 days post-inoculation /1 week	NO levels, Observation of facial muscle movements	Nitric oxide (NO) produced by inducible NO synthase (iNOS) plays a crucial role in HSV-1-induced facial palsy	Before the onset of facial palsy, there was no significant difference in nitric oxide (NO) levels between the HSV-1-inoculated side and the control side of the facial nerve. However, when facial palsy developed, typically around 7 days post-inoculation, the NO levels were significantly elevated on the inoculated side. After recovery from palsy, the NO levels on the inoculated side decreased. Importantly, mice that did not develop transient facial palsy showed no increase in NO levels. The administration of edaravone significantly reduced the incidence of facial palsy in the mouse model /NO, which is produced by inducible NO synthase, plays a crucial role in the onset of facial palsy caused by HSV-1 infection. The free radical scavenger, edaravone, may effectively prevent facial nerve paralysis by mitigating the effects of increased NO levels, highlighting its therapeutic potential for managing HSV-1-related facial nerve damage
Takeda et al. (2008)	Ischemia	Guinea pigs (n = 53) - E0 (Control) group (n = 20): no treatment - E1 group (n = 21): edaravone injections - E2 group (n = 12): edaravone injections began 2 days	Edaravone: 5 mg/kg/day, intraperitoneally for 1 week	Evaluated over 2 to 4 weeks post-injury /28 days	Behavioral test, Histology	Edaravone is a potent free radical scavenger that reduces ROS production, thereby protecting nerve tissues	Edaravone significantly reduced the ROS signal (fluorescence intensity) in the facial nerve, suggesting decreased oxidative stress. This agent decreased the incidence and severity of ischemia-induced facial palsy, particularly when administered immediately after artery interruption, and prevented degenerative changes in the facial nerve, as observed through light and electron microscopy /Edaravone effectively suppresses ROS production and protects against mild to moderate ischemia-induced facial palsy. This agent could be a potential therapeutic option for acute peripheral facial palsy in humans, as it reduced nerve damage even when administered after the onset of symptoms

Abbreviations: EMG, electromyography; CMAP, compound muscle action potential; MUAP, motor unit action potential; NOX2, nicotinamide adenine dinucleotide phosphate oxidase 2; NF200, neurofilament 200; MBP, myelin basic protein; LA, lipoic acid; MP, methylprednisolone; ALA, alpha lipoic acid; CNX, connexin; H&E, hematoxylin and eosin; TUNEL, terminal uridine deoxyribonucleotide labeling; NAC, N-acetylcysteine; PEG, polyethylene glycol; AG, aminoguanidine; MP, methylprednisolone; MeCbl, methylcobalamin; TEM, transmission electron microscope; CMAP, compound muscle action potential; GBE, ginkgo biloba extract; FMN, facial motor nucleus; RSV, rosuvastatin; HHV7, human herpesvirus 7; MDA, malondialdehyde; SOD, superoxide dismutase; GSH, glutathione; PBS, phosphate-buffered saline; HSV-1, herpes simplex virus type 1; NO, nitric oxide; PSELT, SELENOT mimetic peptide; MUAP, motor unit action potential; ROS, reactive oxygen species; CAPE, caffeic acid phenethyl ester.

#### 3.2.1 Coenzyme Q10 (CoQ10)

Coenzyme Q10 (CoQ10) plays a crucial role in generating ATP through the electron transport chain within mitochondria, and ATP is essential for maintaining cellular functions. CoQ10 acts as a potent antioxidant, helping to scavenge ROS within cells. This reduces cellular damage and oxidative stress, prevents lipid peroxidation and DNA damage, and protects cell and mitochondrial membranes from damage. CoQ10 also possesses anti-inflammatory properties that can reduce inflammatory responses and prevent additional inflammation-induced damage following nerve injury, thereby promoting nerve regeneration and accelerating nerve function recovery. After PNI, CoQ10 was shown to promote nerve regeneration and reduce oxidative stress and inflammation (Vachirarojpisan et al., 2024). Wistar rats administered CoQ10 following sciatic nerve injury exhibited faster nerve regeneration, with increased numbers and diameters of regenerated nerve fibers (Moradi et al., 2014). Following compression-induced FNI, CoQ10 administration increased myelin thickness, reduced secondary nerve damage due to oxidative stress, and improved both functional and histological recovery of the injured facial nerve (Yildirim et al., 2015). These results suggest that CoQ10 administration can improve peripheral facial nerve damage by suppressing oxidative stress and reducing inflammation.

#### 3.2.2 Lipoic acid (LA) and alpha-lipoic acid (ALA)

Lipoic acid (LA) and alpha-lipoic acid (ALA) are potent antioxidants that help neutralize free radicals and reduce oxidative stress. These properties are crucial in managing nerve injuries, as oxidative stress can exacerbate nerve damage and impede recovery. LA has been shown to have neuroprotective effects, contributing to maintaining nerve function and promoting nerve regeneration. In diabetic neuropathy, LA administration improved neuropathic symptoms by reducing oxidative stress (Nagamatsu et al., 1995), LA and methylprednisolone (MP) play complementary roles in treating facial nerve injury (FNI), with combination treatment yielding better outcomes in terms of recovering nerve function and reducing inflammation (Tekdemir et al., 2018). Additionally, ALA prevents further damage following peripheral facial nerve transection injury by reducing oxidative stress through the removal of excessive ROS (Yoo et al., 2022). However, conflicting evidence exists regarding the efficacy of ALA. A Cochrane review concluded that ALA treatment in diabetic peripheral neuropathy (DPN) patients has little or no effect on neuropathy symptoms and functional impairment compared to placebo after 6 months of treatment (Baicus et al., 2024). This finding suggests that while ALA has shown potential benefits in some studies, its overall effectiveness in DPN may be limited. The conflicting results highlight the complexity of ALA’s action and suggest that its efficacy may vary depending on factors such as dosage, duration of treatment, and patient population. These inconsistencies indicate the need for further research to determine the optimal use of ALA and LA in nerve injury management and to clarify their roles as therapeutic agents in conditions associated with oxidative stress.

#### 3.2.3 Methylprednisolone (MP)

Methylprednisolone (MP) is a potent corticosteroid known for its ability to reduce inflammation and edema around damaged nerves, thereby promoting nerve healing. By decreasing inflammation, MP can reduce axonal degeneration and promote axonal regeneration, facilitating the overall recovery of nerve function. It is particularly effective in treating various nerve injuries, such as facial nerve palsy, and is widely used in clinical settings (Tekdemir et al., 2018). However, the use of MP is not without controversy. Some studies suggest that MP might have negative effects on nerve regeneration, potentially due to its interference with other physiological processes necessary for nerve recovery (Karlidag et al., 2012). For instance, in a traumatic peripheral facial nerve injury model, MP inhibited the enhancement of collagen fiber without enhancing Schwann cell proliferation or overall nerve function (Yanilmaz et al., 2015). Despite these concerns, MP administration significantly reduces edema and inflammation at the nerve injury site, relieving pressure on the nerve and promoting recovery. MP has shown effectiveness in promoting recovery from facial nerve compression injuries, contributing to functional and structural recovery through its neuroprotective and antioxidant effects (Şeneldir et al., 2024). It significantly improves axonal degeneration, vascular congestion caused by facial nerve palsy, and enhances electrophysiological recovery (Dincer et al., 2025). MP can thus be a useful therapeutic agent in managing nerve injuries by effectively promoting nerve recovery and contributing to functional and structural recovery through its neuroprotective and antioxidant effects (Gölcük and Şeneldir, 2025). MP can thus be a useful therapeutic agent in managing nerve injuries by effectively promoting nerve recovery and contributing to functional and structural recovery through its neuroprotective and antioxidant effects. The Second National Spinal Cord Injury Study (NASCIS-II) study on methylprednisolone for acute spinal cord injury was that methylprednisolone therapy, when administered within 8 h of injury, did not improve motor score recovery in patients with acute cervical or thoracic traumatic spinal cord injury and was associated with a higher rate of complications (Evaniew et al., 2015). Thus, while MP can be a useful therapeutic agent in managing nerve injuries by promoting nerve recovery and contributing to functional and structural recovery, its application should be carefully considered, taking into account the potential for complications and the mixed evidence regarding its efficacy.

#### 3.2.4 Dexamethasone

Dexamethasone is a glucocorticoid that reduces inflammation and suppresses the immune response by inhibiting the release of pro-inflammatory cytokines and mediators. This reduction in inflammation minimizes secondary damage to nerve tissue post-injury, thereby promoting healing and regeneration. By reducing inflammation, dexamethasone also decreases edema around the damaged nerve to improve the microenvironment and facilitate the supply of essential nutrients and oxygen, which are crucial for the nerve recovery process. Although dexamethasone is not primarily recognized as an antioxidant, its ability to control inflammation and stabilize the cellular environment provides effective neuroprotection following nerve injury. For instance, following sciatic nerve injury, dexamethasone has been shown to reduce tissue edema and demyelination, promote remyelination, and help improve gastrocnemius muscle atrophy (Feng and Yuan, 2015). Another study demonstrated that dexamethasone administration after facial nerve compression injury improved functional recovery, as evidenced by increased amplitude values in EMG. In FNI caused by transection, dexamethasone exhibited anti-inflammatory effects, reducing edema and inflammation (Edizer et al., 2019). The safety of low-dose, preservative-free perineural dexamethasone suggests potential neuroprotective effects when used as an adjuvant to local anesthetics. However, concerns are raised about potential neurotoxicity, especially when it is combined with local anesthetics at higher concentrations or when formulations contain sulfites. Additionally, the review highlights the need for caution in patients with preexisting neuropathies due to their increased susceptibility to ischemic damage (De Cassai et al., 2025). These findings collectively indicate that dexamethasone can promote regeneration and recovery after nerve injury through its anti-inflammatory and neuroprotective effects. Nonetheless, clinical use should be carefully considered, especially regarding dosage and patient population, to mitigate potential risks associated with its use.

#### 3.2.5 Ebselen

Ebselen is a neuroprotective agent that protects cellular components from oxidative damage by modulating enzyme cofactors, metalloproteins, and gene expression, exhibiting antioxidant and anti-inflammatory effects, and regulating the immune system. Ebselen acts as an analogue of glutathione peroxidase, an enzyme that neutralizes ROS and reduces oxidative stress. These properties enable ebselen to protect nerve cells from oxidative damage, which is a common result of nerve injury. In animal models of spinal cord injury, ebselen administration reduced oxidative stress due to spinal cord injury and increased the levels of antioxidant enzymes, thereby protecting neurons (Kalayci et al., 2005). After ischemia/reperfusion injury, ebselen inhibited excessive glutamate release and enhanced the inhibitory effects of GABA to alleviate nerve damage (Seo et al., 2009). Additionally, following peripheral facial nerve compression injury, ebselen administration yielded recovery patterns (Şeneldir et al., 2024). These findings indicate that ebselen exhibits neuroprotective effects in various nerve injury models, aiding in the protection and regeneration of nerve cells by reducing oxidative stress and exerting anti-inflammatory and antioxidant actions.

#### 3.2.6 Oxytocin

Oxytocin is a hormone essential for social bonding, childbirth, and lactation; it is produced in the hypothalamus and secreted by the pituitary gland. Beyond its well-known role in enhancing social interactions and maternal behaviors, oxytocin also reduces oxidative stress through powerful antioxidant actions by increasing the activity of antioxidant enzymes such as SOD and glutathione peroxidase, which neutralize harmful ROS. Oxytocin also reduces lipid peroxidation to help maintain cell membrane integrity, lowers inflammation, and reduces oxidative stress to protect cells from oxidative damage. In a sciatic nerve transection injury model, oxytocin administration resulted in higher amplitudes and shorter latency times in EMG, with increased vascular diameter and thickness, suggesting that it promotes revascularization and improves functional recovery (Gümüs et al., 2015). Oxytocin administration can also promote nerve recovery by upregulating nerve growth factor (NGF), Nrf2, and irisin (Tosyalı et al., 2023). Following facial nerve compression injury, oxytocin administration effectively modulated apoptosis and promoted facial nerve regeneration to yield significant improvements in functional recovery (Tanyeri et al., 2015). These findings suggest that oxytocin has potential as a therapeutic agent that may promote nerve regeneration and recovery through its neuroprotective and anti-inflammatory actions following PNI.

#### 3.2.7 Resveratrol

Resveratrol is a well-known polyphenolic compound; it is found in various plants, especially grapes, red wine, berries, and peanuts, and has antioxidant, anti-inflammatory, and cardioprotective effects. In PNI, in particular, resveratrol contributes to reducing oxidative stress by neutralizing ROS. Resveratrol increases the activity of antioxidant enzymes in the body, reduces inflammation, and promotes mitochondrial function. In a sciatic nerve compression injury model, resveratrol administration increased the protein expression of vascular endothelial growth factor (VEGF) and the number of axons in the damaged nerve, and thus enhanced nerve regeneration (Ding et al., 2018). Resveratrol also promoted nerve recovery by accelerating myelin clearance through the autophagy of Schwann cells (Zhang et al., 2020). Following facial nerve compression injury, resveratrol administration significantly improved blink reflex recovery and functional recovery, as evidenced by increased amplitudes in EMG In particular, resveratrol modulated apoptosis and increased intercellular levels of connexins (connexin 32 and 43), which are important for cell communication and tissue integrity during the healing process, and thus aided nerve recovery (Tanyeri et al., 2015). These findings suggest that resveratrol can play an important role in promoting recovery after nerve injury.

#### 3.2.8 Melatonin

Melatonin is a hormone that is primarily produced by the pineal gland in the brain. It is well known to regulate the sleep-wake cycle, but it also possesses powerful antioxidant, anti-inflammatory, and neuroprotective effects. Based on these properties, extensive research has examined melatonin’s potential therapeutic effects in various conditions, including nerve injury. Melatonin reduces oxidative stress by stimulating antioxidant enzymes, such as SOD, catalase (Ct), and peroxidase (Uyanikgil et al., 2017). Schwann cells express melatonin receptors to enable melatonin signaling. Melatonin promotes the reorganization, migration, dedifferentiation, and regeneration of Schwann cells (Klymenko and Lutz, 2022). In promoting peripheral nerve recovery, melatonin inhibits ROS generation through mitophagy, maintains autophagic flow, and suppresses mitochondrial apoptosis [48]. After FNI, intraperitoneal injection of melatonin yielded more extensive systemic effects and better preservation of nerve diameter and myelin thickness compared to controls or local administration. Melatonin also increased the activity of antioxidant enzymes, such as SOD and glutathione peroxidase, to further protect cells from oxidative damage (Edizer et al., 2019). Following traumatic peripheral FNI, melatonin administration reduced the collagen fiber increase and myelin degeneration seen in controls, and thus enhanced nerve regeneration (Yanilmaz et al., 2015). Melatonin shows potential as a neuroprotective agent that can promote recovery following PNI. However, structure–function translation has been inconsistent in severe injury settings. In an axotomy/anastomosis model, melatonin yielded superior histopathology but did not produce significant short-term electrophysiological gains (e.g., EMG amplitude/latency) over a 12-week window, underscoring model- and dose-contingent effects on function. Comparative data in transection contexts further suggest that while melatonin can outperform steroids on select structural or electrophysiological parameters, near-term functional improvements are not uniformly observed and may require longer follow-up to emerge. Taken together, melatonin shows promise as an experimental antioxidant/neuroprotective agent in FNI, with benefits influenced by injury severity, timing, and dose. Translation will require standardized functional endpoints (EMG, reflexes, conduction), formal dose–response evaluation, and extended follow-up to determine whether structural gains consistently convert into clinically meaningful functional recovery.

#### 3.2.9 N-acetylcysteine (NAC)

NAC is a medication and dietary supplement derived from L-cysteine, known for its powerful antioxidant properties and its use as a mucolytic agent in respiratory diseases. NAC acts as a precursor for glutathione (GSH) in the body, playing a crucial role in regulating intracellular oxidative stress by increasing intracellular concentrations of GSH. Additionally, NAC inhibits the activation of nuclear factor kappa B (NF-κB), thereby reducing the levels of inflammatory cytokines such as TNF-α and interleukins (IL-6, IL-1β) (Tenório et al., 2021). Following sciatic nerve compression injury, NAC administration reduced perineurial thickness, axonal degeneration, axonal lysis, edema, inflammation, muscle atrophy, and myopathy through its antioxidant effects (Sarıtaş et al., 2024). In the context of facial nerve injuries, NAC has been shown to improve nerve regeneration and reduce nerve degeneration by increasing the proliferation of Schwann cells and modulating glial cell activity (Karlidag et al., 2012). NAC likely influences glial activation by reducing oxidative stress and inflammation, which can help prevent the overactivation of glial cells such as microglia and astrocytes. This modulation can decrease the release of neurotoxic mediators and promote a more supportive environment for nerve repair. In rats with compression-induced facial nerve injury (FNI), NAC administration resulted in better recovery in terms of eye blinking and vibrissae movement compared to controls at both 2 and 4 weeks post-injury. Electromyography (EMG) measurements indicated greater amplitude recovery, with eye-blink function recovering more rapidly by week 2 and vibrissae function by week 4 (Rivera et al., 2017). These results suggest that NAC can enhance the functional and electrical recovery of nerves following injury, in part by modulating glial cell responses to create a more favorable environment for nerve regeneration.

#### 3.2.10 Polyethylene glycol (PEG)

PEG is a highly biocompatible and soluble compound that is widely used in the medical field. In FNI, it functions by fusing damaged axonal membranes to restore axonal continuity, enhance nerve conduction and functional recovery, and restore action potentials at the injury site. PEG also helps maintain nerve function by preventing Wallerian degeneration, which is the degeneration of the axonal portion of a neuron upon its separation from the cell body (Smith et al., 2025). The application of PEG solution to the sciatic nerve at the cut axonal end was found to preserve neuromuscular junctions and prevent muscle atrophy, leading to the recovery of sensory and motor functions. This so-called “PEG fusion” provides a rapid and effective method for PNI repair, yielding significantly better functional outcomes compared to traditional nerve suturing techniques (Ghergherehchi et al., 2019). After sciatic nerve transection, PEG administration yielded functional recovery in EMG and inclined plane tests, increased levels of nerve growth factor (NGF), reduced fibrosis, and increased axonal density, indicating that it promotes neuroprotection and regeneration (Tunç et al., 2025). In a study involving transection of the mandibular branch of the facial nerve, the PEG fusion group exhibited larger axonal diameters compared to the control group, indicating that PEG fusion had a positive impact on preserving axonal structure (Salomone et al., 2018). These results suggest that PEG can promote functional and structural recovery following peripheral nerve injury.

#### 3.2.11 Aminoguanidine (AG)

Aminoguanidine (AG) is a compound known for its potent antioxidant effects and its ability to inhibit the formation of advanced glycation end-products (AGEs) and inducible nitric oxide synthase (iNOS). AG also demonstrated antioxidant activity against brain damage induced by doxorubicin (DOX), reducing levels of malondialdehyde (MDA) and glutathione peroxidase (GPx) while increasing the activity of glutathione S-transferase (GST) [54]. The increase in oxidative stress due to ischemia was mitigated by AG treatment, which increased the oxidized glutathione ratio (GSH/GSSG), activated GST, and reduced lipid peroxidation (Pasten et al., 2021). After ischemia was induced by occlusion of the right common iliac artery and femoral artery, AG administration was associated with functional recovery in hindlimb function tests, reduced endoneurial edema, and decreases in ROS and apoptosis (Alipour et al., 2011). Following traumatic peripheral FNI, AG administration reduced myelin degeneration and myelin debris accumulation, and EMG showed increases in nerve conduction velocity and amplitude over time, indicating improved nerve function (Yanilmaz et al., 2015). In PNI, AG decreases ROS and reduces the production of NO, which can form harmful peroxynitrite when combined with superoxide; thus, AG reduces oxidative stress in this context. Together, these results indicate that AG promotes nerve recovery and functional improvement by protecting nerve cells and maintaining structural integrity after injury.

#### 3.2.12 Methylcobalamin (MeCbl)

Methylcobalamin (MeCbl), the active form of vitamin B12, is essential for nerve health and effective in promoting nerve regeneration after PNI. MeCbl enhances the body’s antioxidant defenses to neutralize harmful ROS and contribute to reducing oxidative stress. Following sciatic nerve compression, MeCbl administration resulted in recovery of motor and sensory functions, as evidenced by improvements in the sciatic function index (SFI) and von Frey filament tests. MeCbl enhanced nerve conduction velocity, promoted nerve regeneration, and increased myelination to aid in axonal repair (Suzuki et al., 2017). In a rat model of sciatic nerve injury, continuous MeCbl administration via an osmotic mini-pump increased the activities of Erk1/2 and Akt, which are key signaling proteins involved in axon growth and neuron survival, and thereby promoted recovery of motor and sensory functions (Okada et al., 2010). After facial nerve compression injury, MeCbl promoted nerve regeneration and further reduced oxidative stress; its effects were particularly pronounced when combined with silicone-based drugs (Koyama et al., 2022). These findings demonstrate that MeCbl is effective in managing oxidative stress and facilitating nerve repair.

#### 3.2.13 Si-based agents

Silicon-based agents react with water to produce hydrogen gas, which acts as a selective antioxidant by targeting and neutralizing nerve cell-damaging ROS, hydroxyl radicals, and peroxynitrite. These agents exert anti-inflammatory effects by reducing pro-inflammatory cytokines and factors, show anti-apoptotic effects by inhibiting pathways leading to cell death, and yield anti-fibrotic effects by decreasing fibrosis-related markers (Koyama et al., 2023). In the case of PNI, oxidative stress due to excessive ROS can hinder nerve regeneration. Silicon-based agents protect nerve cells and create a more favorable environment for nerve regeneration by reducing oxidative stress, making them effective in promoting recovery from nerve damage-related conditions such as facial paralysis. After compression-induced FNI, silicon-based agents improved nerve regeneration by promoting myelination and reducing oxidative stress (Koyama et al., 2022). Due to these properties, silicon-based agents are reported to contribute to the treatment of nerve injuries.

#### 3.2.14 Ozone therapy

Ozone therapy involves the use of ozone gas to stimulate the body’s natural antioxidant defense system, inducing appropriate oxidative stress to exert anti-inflammatory effects, eradicate pathogenic microorganisms, and promote tissue regeneration. Due to its small molecular structure, ozone quickly penetrates cells and tissues to eliminate pathogens and generate oxygen, promoting cellular and tissue recovery. Ozone therapy is effective in alleviating neuropathic pain by promoting efferocytosis of macrophages and activating AMPK/Gas6-MerTK/SOCS3 signaling to reduce neuroinflammation. In a chronic constriction injury (CCI) model, ozone therapy alleviated pain through this mechanism (Ruan et al., 2024). Following sciatic nerve transection, ozone therapy improved function in the SFI and withdrawal reflex (WDR) by enhancing antioxidant enzyme activity, promoting nerve fiber myelination, and increasing functional recovery (Ogut et al., 2020). After compression-induced FNI, ozone therapy improved damage to myelinated axons and demonstrated beneficial effects on regeneration (Ozbay et al., 2017). These results suggest that ozone therapy is effective in reducing oxidative stress associated with PNI and promoting nerve regeneration.

#### 3.2.15 Rosuvastatin

Rosuvastatin is primarily used to lower cholesterol levels, but it also possesses antioxidant properties that may be beneficial in treating PNI. Additionally, rosuvastatin enhances the body’s natural antioxidant defenses, improves endothelial function, and reduces inflammation, thereby creating a favorable environment for nerve healing and regeneration. Following sciatic nerve compression injury, rosuvastatin administration increased motor function recovery in the SFI test, increased the amplitude of compound muscle action potentials (CMAP), and improved nerve conduction. It also increased the number and thickness of myelinated fibers and enhanced the expression levels of neurotrophic factors, such as nerve growth factor (NGF) and brain-derived neurotrophic factor (BDNF), to promote nerve cell function and recovery (Abdolmaleki et al., 2020). In a peripheral facial paralysis animal model, rosuvastatin administration contributed to electrophysiological recovery and significantly improved axonal degeneration and vascular congestion, and thus had beneficial effects on facial nerve regeneration (Dincer et al., 2025). In sum, rosuvastatin has shown potential as a therapeutic agent in the treatment of PNI.

#### 3.2.16 Ginkgo biloba extract (GBE)

Ginkgo biloba extract (GBE) is derived from the leaves of the ginkgo biloba tree and is known for its medicinal properties. It contains flavonoids and terpenoids, which have potent antioxidant and anti-inflammatory effects and help protect vascular endothelial cells from oxidative damage by scavenging free radicals (Singh et al., 2019). GBE also promotes microcirculation, which is crucial for nerve health and recovery. The extensive antioxidant properties of GBE may be beneficial in the prevention and treatment of diseases associated with free radical-induced damage (Rong et al., 1996). In an animal model of compression-induced facial nerve injury, recovery of vibrissae function was faster in the GBE-treated group compared to controls, and there were significant improvements in muscle action potential thresholds and conduction velocity in the GBE group (Jang et al., 2012). This suggests that GBE treatment facilitates electrophysiological recovery of the injured facial nerve.

#### 3.2.17 Vitamin E

Vitamin E is a fat-soluble vitamin primarily known for the antioxidant activity of its α-tocopherol form. It is particularly effective in inhibiting lipid peroxidation, thereby protecting cell membranes from oxidative stress. Vitamin E also reduces oxidative stress by scavenging ROS, preventing nerve cell degeneration. These protective actions lead to reduced inflammation and enhanced recovery, making this vitamin a potentially useful therapeutic agent for diseases related to nerve damage. Vitamin E has demonstrated beneficial effects on motor function, pain hypersensitivity, and histopathological changes after nerve injury, promoting neuroprotection and regeneration by inhibiting oxidative stress pathways (Tamaddonfard et al., 2014). In a model of transection-induced FNI, vitamin E significantly increased the survival rate of motor neurons in the facial nucleus of the CNS and demonstrated neuroprotective effects by reducing lipid peroxidation and oxidative stress (Hoshida et al., 2009). However, the effects of vitamin E are not universally positive. A study on the nematode *Caenorhabditis elegans* revealed that high concentrations of vitamin E (400 μg/mL) can adversely affect thermosensation and thermotaxis learning. The study showed that such high doses impaired presynaptic function and decreased the fluorescent intensities of key neurons involved in thermosensation and learning. These findings suggest that while vitamin E has significant neuroprotective properties, its administration at high concentrations can lead to adverse effects, particularly on neuronal development and function (Li et al., 2013). These protective actions of vitamin E, when administered at appropriate doses, may notably promote neuroprotection and regeneration by inhibiting pathways related to oxidative stress following nerve injury. Benefits are dose- and context-dependent. Moderate dosing has been associated with functional and histological improvements, whereas high exposures have produced paradoxical or adverse effects (e.g., increased gliosis or neuronal dysfunction in select models), underscoring the need for formal dose–response evaluation and safety monitoring. Accordingly, clinical translation will require standardized functional endpoints and careful dose-finding before recommendations can be made.

#### 3.2.18 Caffeic acid phenethyl ester (CAPE)

CAPE is a bioactive compound that is found in bee propolis and known for its anti-inflammatory, antioxidant, and neuroprotective properties. As an antioxidant, CAPE neutralizes free radicals, inhibits lipid peroxidation, enhances antioxidant enzyme activity, and reduces oxidative stress and inflammation by inhibiting the NF-κB pathway. After spinal cord injury, CAPE downregulated DRP1 while activating SIRT1 and PGC1α to alleviate mitochondrial dysfunction and reduce inflammation and oxidative stress (Zhang et al., 2024). CAPE activates the Nrf2 pathway to increase antioxidant enzyme expression, stabilize ROS, and protect nerve cells. CAPE also inhibits the NF-κB pathway, thereby reducing pro-inflammatory cytokine expression and preventing neuroinflammation (Pérez et al., 2023). In a facial nerve crush model, CAPE administration promoted nerve regeneration; when it was combined with MP functional recovery was faster, as measured in terms of corneal reflex. Histopathological evaluation of the CAPE + MP group showed lower levels of axonal degeneration and vascular congestion, with thicker myelin sheaths (Gölcük and Şeneldir, 2025). These findings suggest that CAPE, with its neuroprotective, antioxidant, and anti-inflammatory effects, could serve as a potential therapeutic alternative that could be used to reduce the dosage and mitigate the systemic side effects of MP.

#### 3.2.19 SELENOT mimetic peptide (PSELT)

SELENOT mimetic peptide (PSELT), which is a synthetic peptide derived from the selenoprotein, SELENOT, is known for its antioxidant and neuroprotective properties. PSELT effectively reduces oxidative stress in PNI by neutralizing ROS, enhancing endogenous antioxidant defenses, and preventing lipid peroxidation. Additionally, PSELT modulates protective signaling pathways and reduces inflammation to create a favorable environment for nerve regeneration and functional recovery. PSELT administration improved motor coordination and spontaneous motor activity in mice treated with MPTP by targeting oxidative stress and regulating gene expression to promote cell survival (Alsharif et al., 2021). Following facial nerve transection, PSELT administration enhanced motor recovery, with higher mean motor unit action potential amplitude and shorter duration, with increased axonal density and myelination, and thus improved nerve regeneration and functional recovery (Pothion et al., 2022). Through its antioxidant and neuroprotective actions, PSELT can regulate key signaling pathways and reduce oxidative stress and inflammation, thereby enhancing nerve regeneration and functional recovery.

#### 3.2.20 Edaravone

**FIGURE 2:**
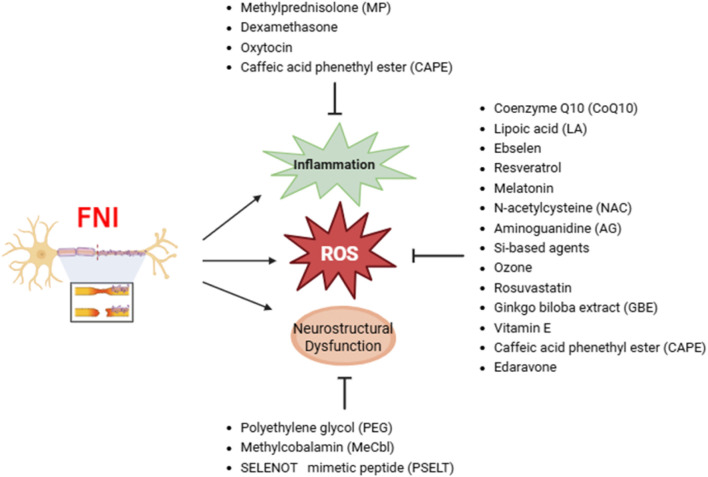
Antioxidant interventions in FNI.

Edaravone is a potent antioxidant that acts as a FR scavenger, effectively neutralizing unstable molecules that cause oxidative stress and cellular damage. By removing ROS and other FR, edaravone protects nerve cells from oxidative damage, reduces inflammation, and promotes nerve recovery. Due to its antioxidant and anti-inflammatory effects, edaravone has been clinically used in treating acute cerebral ischemia and amyotrophic lateral sclerosis (ALS). In a placental ischemia model, it reduced brain inflammation and oxidative stress, and improved fetal survival, growth, and maternal organ function (Yamashita and Abe, 2024). Edaravone decreased oxidative stress markers such as malondialdehyde (MDA), increased the levels of antioxidants such as SOD and GSH/GSSG, and reduced neuroinflammation through decreased activation of hippocampal microglia (Iba1+ cells) (Ma et al., 2024). However, not all studies report positive outcomes with edaravone. Some research suggests that supraphysiologic doses may lead to delayed recovery or adverse effects. It is crucial to consider the dose–response relationship, as varying doses can significantly impact the therapeutic outcomes. Additionally, while edaravone’s ability to reduce oxidative stress is effective in treating nerve damage-related conditions, such as Bell’s palsy caused by HSV-1 infection, potential off-target effects, particularly at high doses, should be addressed (Hato et al., 2013). Given its strong antioxidant and anti-inflammatory properties, edaravone shows potential as an effective therapeutic agent for preventing damage to cell membranes and tissues in conditions such as ischemic nerve injury (Takeda et al., 2008). Nonetheless, further research is needed to fully understand the optimal dosing and potential risks associated with its use in different clinical scenarios (Table 3) (Figure 2).

**TABLE 3:** Classification of antioxidants and their roles in facial nerve injury recovery.

Group	Antioxidant	Primary action
Direct ROS Scavenging & Antioxidant Enzyme Activation	Coenzyme Q10 (CoQ10)	Mitochondrial electron transport, ROS removal, ATP generation
	Lipoic acid (LA)	Free radical neutralization, neuroprotection
	Ebselen	Acts as glutathione peroxidase, ROS reduction
	Resveratrol	ROS neutralization, antioxidant enzyme activation, mitochondrial support
	Melatonin	Antioxidant and anti-inflammatory effects, antioxidant enzyme activation
	N-acetylcysteine (NAC)	Glutathione precursor, oxidative stress reduction, anti-inflammatory effects
	Aminoguanidine (AG)	ROS neutralization, NOS reduction
	Si-based agents	ROS neutralization via hydrogen gas production
	Ozone	Antioxidant defense stimulation through mild oxidative stress
	Rosuvastatin	ROS level reduction, enhancement of natural antioxidant defenses
	Ginkgo biloba extract (GBE)	Antioxidant effects via flavonoids and terpenoids
	Vitamin E	Lipid peroxidation suppression, cell membrane protection
	Caffeic acid phenethyl ester (CAPE)	Free radical neutralization, antioxidant enzyme activation
	Edaravone	Potent ROS scavenging
Anti-inflammatory & Microenvironment Improvement	Methylprednisolone (MP)	Inflammation and edema reduction, corticosteroid effects
	Dexamethasone	Strong anti-inflammatory effects, cytokine release and edema reduction
	Oxytocin	Antioxidant and anti-inflammatory effects
	Caffeic acid phenethyl ester (CAPE)	Anti-inflammatory properties alongside antioxidant effect
Neurostructural & Functional Recovery Promotion	Polyethylene glycol (PEG)	Axonal membrane fusion aid for nerve continuity (mechanical repair)
	Methylcobalamin (MeCbl)	Nerve regeneration promotion, apoptosis prevention
	SELENOT mimetic peptide (PSELT)	Protective signaling pathway activation, cell survival and growth promotion

### 3.3 Antioxidants effective against FNI

#### 3.3.1 Crush/compression models

In crush/compression models, several antioxidants improved functional readouts. Resveratrol, dexamethasone, and oxytocin shortened reflex recovery times and increased EMG amplitudes versus controls; ebselen achieved functional and histological recovery comparable to methylprednisolone, suggesting a potential steroid-sparing alternative. CoQ10 lowered stimulation thresholds and increased myelin thickness with favorable histology, and Ginkgo biloba extract accelerated vibrissae function recovery with improved conduction metrics. Given the intrinsically higher regenerative potential and substantial spontaneous recovery in crush injuries, these gains are interpreted with caution and not extrapolated to more severe models.

#### 3.3.2 Transection/anastomosis models

In transection/anastomosis settings, structural improvements often outpaced short-term functional gains. For example, melatonin yielded superior histopathology without significant EMG improvement within 12 weeks. N-acetylcysteine outperformed methylprednisolone after anastomosis, with better EMG and histology and less degeneration, underscoring the risk of extrapolating steroid benefits across models. SELENOT-mimetic peptides (PSELT) improved MUAP characteristics and axonal metrics, suggesting mechanistic promise that warrants longer functional follow-up. PEG-fusion mitigated demyelination and increased axonal diameter but provided limited functional enhancement over the study window. Comparator data further indicate advantages over steroids in select parameters: aminoguanidine and melatonin showed superior electrophysiological and structural profiles to methylprednisolone in transection settings, although short-term functional gains were not always consistent.

#### 3.3.3 Ischemic/viral models

In ischemic/viral contexts, edaravone reduced oxidative stress markers and suggested clinical benefit; however, assessments often relied on clinical observation without standardized electrophysiology. Standardized head-to-head confirmation against both no-treatment and steroid comparators is needed to define its incremental effect size and dose window (Table 4).

**TABLE 4:** Model-stratified summary of antioxidant effects in FNI.

Models	Agents	Direction of effects
Crush/Compression	Resveratrol; Dexamethasone; Oxytocin	Improved reflex recovery and EMG vs. control
	CoQ10	Lower thresholds; thicker myelin; better histology
	Ginkgo biloba extract	Faster functional/electrophysiological recovery
Transection/Anastomosis	Melatonin	Histological improvement without significant EMG gains
	NAC vs. MP	NAC > MP for regeneration; MP associated with greater degeneration
	PSELT	Improved MUAP profile and histology
Ischemic/viral	Edaravone	Reduced oxidative stress; clinical benefit suggested

### 3.4 Clinical and translational implications

#### 3.4.1 Crush/compression

Several antioxidants (e.g., resveratrol, dexamethasone, oxytocin, ebselen, coenzyme Q10, and Ginkgo biloba extract) were associated with earlier improvements in electrophysiological or behavioral readouts versus controls in crush/compression models. Given the intrinsically high spontaneous recovery in these injuries, such gains should be interpreted as supportive signals rather than definitive efficacy and should not be extrapolated to more severe injuries without confirmatory data. Coenzyme Q10 and Ginkgo biloba extract, for instance, improved conduction metrics and myelination in preclinical studies, suggesting potential as adjuncts pending standardized endpoints and human validation.

#### 3.4.2 Transection/anastomosis

In transection/anastomosis settings, structural improvements (e.g., myelination, axonal caliber) frequently outpaced near-term functional recovery. In some comparisons, N-acetylcysteine showed advantages over steroids on EMG and histology; SELENOT-mimetic peptides (PSELT) and PEG-fusion improved axonal metrics and reduced demyelination, yet provided limited short-window functional enhancement—indicating the need for longer follow-up and standardized electrophysiological endpoints before clinical recommendations can be made. Melatonin yielded histopathologic benefits with inconsistent short-term EMG gains, underscoring model- and dose-contingent translation challenges.

#### 3.4.3 Infectious/ischemic FNI

In infectious/ischemic contexts, edaravone reduced oxidative stress markers with suggested clinical benefit; however, assessments often relied on clinical observation without standardized electrophysiology. Head-to-head confirmation against both no-treatment and steroid comparators is required to define incremental effect size and dosing windows.

#### 3.4.4 Degenerative/metabolic contexts (aging, diabetes, vascular disease)

Most included studies used young, otherwise healthy animals. Because nerve regenerative capacity declines with age and comorbidities elevate oxidative stress, treatment effects may attenuate in older or metabolically compromised patients; dose windows and safety margins may also differ. Translational studies should therefore prespecify age/comorbidity strata and oxidative-stress baselines when designing trials.

### 3.5 Clinician-facing translation and practical use

The model-stratified signals synthesized above can be translated into a pragmatic, clinician-facing framework that outlines how antioxidants and related agents might be used as adjuncts in real-world scenarios, while avoiding overgeneralization from preclinical data. We summarize potential use by injury mechanism, therapeutic timing, route of administration, monitoring strategy, safety considerations, and evidence level. These statements are intended to guide research and shared decision-making rather than to recommend off-label treatment.

#### 3.5.1 Traumatic/compressive injuries (acute crush/compression)

In acute crush or compressive injuries-particularly within the first 72 h when some residual conduction is expected and spontaneous recovery is common-adjunctive antioxidants may be considered alongside standard care to potentially accelerate electrophysiological recovery. Preclinical studies report earlier reflex recovery and higher EMG amplitudes with resveratrol, dexamethasone, and oxytocin; ebselen achieved functional and histological recovery comparable to methylprednisolone, suggesting a possible steroid-sparing role. Coenzyme Q10 lowered stimulation thresholds and increased myelin thickness with favorable histology, and Ginkgo biloba extract accelerated recovery of vibrissae function with improved conduction metrics. Because crush models have intrinsically high spontaneous recovery, any apparent gains should be confirmed with standardized ENoG/EMG, blink reflex, and clinical grading before being interpreted as drug effects. Safety requires attention to bleeding risk with Ginkgo in patients on antithrombotics, potential CYP-mediated interactions with resveratrol, and the hyperglycemia/infection risks of dexamethasone. These signals are supportive but should not be extrapolated to more severe injuries without confirmatory data.

#### 3.5.2 Transection/anastomosis (post-repair)

Following transection and primary repair, structural improvements (myelination, axonal caliber) frequently precede measurable functional gains. In this context, N-acetylcysteine has shown advantages over methylprednisolone on EMG and histology with less degeneration, supporting consideration as an early adjunct after anastomosis rather than defaulting to steroids. Melatonin has yielded histopathologic benefits without consistent short-term EMG improvements, indicating that function may lag behind structure in the early window. SELENOT-mimetic peptides (PSELT) and PEG-fusion have improved axonal metrics and reduced demyelination, respectively, yet have provided limited functional enhancement over short follow-up intervals and remain investigational. Clinically, we suggest planning extended follow-up of at least 6–12 months with MUAP and CMAP analyses and, where available, high-resolution imaging to capture delayed functional convergence. For safety, clinicians should be aware of gastrointestinal intolerance to NAC and rare bronchospasm with intravenous formulations. Comparative signals favoring NAC and, in select parameters, aminoguanidine or melatonin over steroids underscore the need to avoid uncritical extrapolation of steroid benefits across models.

#### 3.5.3 Infectious/ischemic presentations

In ischemic or viral facial palsy, edaravone has reduced oxidative stress markers and, in animal models, decreased the incidence and severity of palsy; however, many assessments have relied on clinical observation rather than standardized electrophysiology. Accordingly, any adjunctive use should be embedded in protocols that include head-to-head comparisons against both no-treatment and steroid controls, with predefined electrophysiological endpoints to quantify incremental benefit, and with renal function monitoring for safety. Until such data are available, edaravone should be regarded as a hypothesis-generating adjunct rather than a routine therapy in these indications.

#### 3.5.4 A pragmatic algorithm for use

A practical approach begins by triaging patients by mechanism (traumatic/compressive, transection/anastomosis, infectious/ischemic) and timing (acute 0–72 h, subacute 3–14 d, chronic >3 mo). Standard care is applied per guidelines, followed by a screen for contraindications and drug–drug interactions. An antioxidant adjunct is considered when preclinical signals match the mechanism of injury and safety is acceptable, recognizing the high spontaneous recovery seen in crush models and the structural–functional lag typical of transection settings. Patients are monitored with ENoG/EMG, blink reflex, and standardized facial scoring over 2–12 weeks, and continuation or de-escalation is determined by predefined thresholds and longitudinal trends. This algorithm prioritizes standardized electrophysiology and longer follow-up in models where short-term functional gains are unlikely to capture the full effect size.

## 4 Conclusion

FNI profoundly affects quality of life. Oxidative stress and consequent mitochondrial dysfunction contribute to impaired nerve recovery. Based on mechanistic and predominantly preclinical evidence (with limited clinical data), our synthesis suggests that antioxidants-including CoQ10, ALA, melatonin, NAC, MeCbl, resveratrol, and others-may mitigate reactive oxygen species and inflammation and support neuroprotection in experimental models. To avoid overgeneralization, we emphasize model-specific differences: crush/compression models more often show early electrophysiological or behavioral gains, whereas transection/anastomosis models frequently exhibit histological improvement without consistent short-term functional recovery; ischemic/viral contexts remain under-evaluated with standardized electrophysiological endpoints. Outcomes also depend on regimen: earlier initiation and appropriate dosing tend to be more favorable in preclinical settings, and select combinations may show additive effects; however, these remain hypothesis-generating. Taken together-with heterogeneity in dosing, timing, formulations, small sample sizes, and inconsistent functional gains-current data do not support clinical recommendations. Direct clinical evidence for specific supplement-drug combinations is scarce, and safety/interaction profiles require systematic evaluation. We therefore refrain from prescriptive guidance and present combination approaches as testable hypotheses. Future work should prioritize well-powered randomized trials in FNI that optimize formulation, dosing, and timing; incorporate biomarker-based stratification and standardized functional and patient-reported outcomes with adequate follow-up; prespecify timing/dose exploration and combination arms; and rigorously monitor adverse events and pharmacologic interactions. Such studies are essential to determine whether antioxidant strategies can be translated into effective, evidence-based care that improves neurological recovery and patients’ quality of life.
